# Transient Elastography-Based Liver Profiles in a Hospital-Based Pediatric Population in Japan

**DOI:** 10.1371/journal.pone.0137239

**Published:** 2015-09-23

**Authors:** Yuki Cho, Daisuke Tokuhara, Hiroyasu Morikawa, Yuko Kuwae, Eri Hayashi, Masakazu Hirose, Takashi Hamazaki, Akemi Tanaka, Tomoyuki Kawamura, Norifumi Kawada, Haruo Shintaku

**Affiliations:** 1 Department of Pediatrics, Osaka City University Graduate School of Medicine, Osaka, Japan; 2 Department of Hepatology, Osaka City University Graduate School of Medicine, Osaka, Japan; 3 Department of Pathology, Osaka City University Graduate School of Medicine, Osaka, Japan; 4 Department of Pediatrics, Izumi Municipal Hospital, Osaka, Japan; Taipei Veterans General Hospital, TAIWAN

## Abstract

**Background & Aims:**

The utility of transient elastography (FibroScan) is well studied in adults but not in children. We sought to assess the feasibility of performing FibroScans and the characteristics of FibroScan-based liver profiles in Japanese obese and non-obese children.

**Methods:**

FibroScan examinations were performed in pediatric patients (age, 1–18 yr) who visited Osaka City University Hospital. Liver steatosis measured by controlled attenuation parameter (CAP), and hepatic fibrosis evaluated as the liver stiffness measurement (LSM), were compared among obese subjects (BMI percentile ≥90%), non-obese healthy controls, and non-obese patients with liver disease.

**Results:**

Among 214 children examined, FibroScans were performed successfully in 201 children (93.9%; median, 11.5 yr; range, 1.3–17.6 yr; 115 male). CAP values (mean±SD) were higher in the obese group (n = 52, 285±60 dB/m) compared with the liver disease (n = 40, 202±62, *P*<0.001) and the control (n = 107, 179±41, *P*<0.001) group. LSM values were significantly higher in the obese group (5.5±2.3 kPa) than in the control (3.9±0.9, *P*<0.001), but there were no significant differences in LSM between the liver disease group (5.4±4.2) and either the obese or control group. LSM was highly correlated with CAP in the obese group (ρ = 0.511) but not in the control (ρ = 0.129) or liver disease (ρ = 0.170) groups.

**Conclusions:**

Childhood obesity carries a high risk of hepatic steatosis associated with increased liver stiffness. FibroScan methodology provides simultaneous determination of CAP and LSM, is feasible in children of any age, and is a non-invasive and effective screening method for hepatic steatosis and liver fibrosis in Japanese obese children.

## Introduction

Introduced commercially in 2002, transient elastography (FibroScan) can be used to evaluate liver stiffness and hepatic fat deposition in the absence of liver biopsy. To date, more than 300 accumulated studies have demonstrated the feasibility and usefulness of FibroScan methodology in adult patients with various liver conditions, including chronic hepatitis B and C, liver transplantation, and non-alcoholic steatohepatitis (NASH) [1–11]. Although non-invasiveness is a key feature of effective evaluation methods not only for adults but also children, few studies have addressed the feasibility and usefulness of FibroScan specifically in pediatric populations [12–16].

One of the most important indications for the use of FibroScan methodology is in the evaluation of non-alcoholic fatty liver disease (NAFLD), which can vary in presentation from simple steatosis to non-alcoholic steatohepatitis (NASH), a leading cause of chronic liver disease in both adults and children [17–26]. The prevalence of NAFLD ranges from 2.6% to 9.6% [20–23] in children overall and from 22.5% to 44.0% in obese children in particular [20,21,24]. Because pediatric NAFLD can progress to cirrhosis, which has serious outcomes if left untreated [27,28], using FibroScan to effectively and non-invasively discriminate between simple fatty liver and NASH in obese children would be beneficial.

In this study, we sought to clarify the feasibility and usefulness of FibroScan in Japanese children, especially focusing on differences in the resulting liver profile (i.e., liver stiffness and hepatic fat deposition) between obese and non-obese pediatric populations.

## Patients and Methods

### Patients

**Fig 1:**
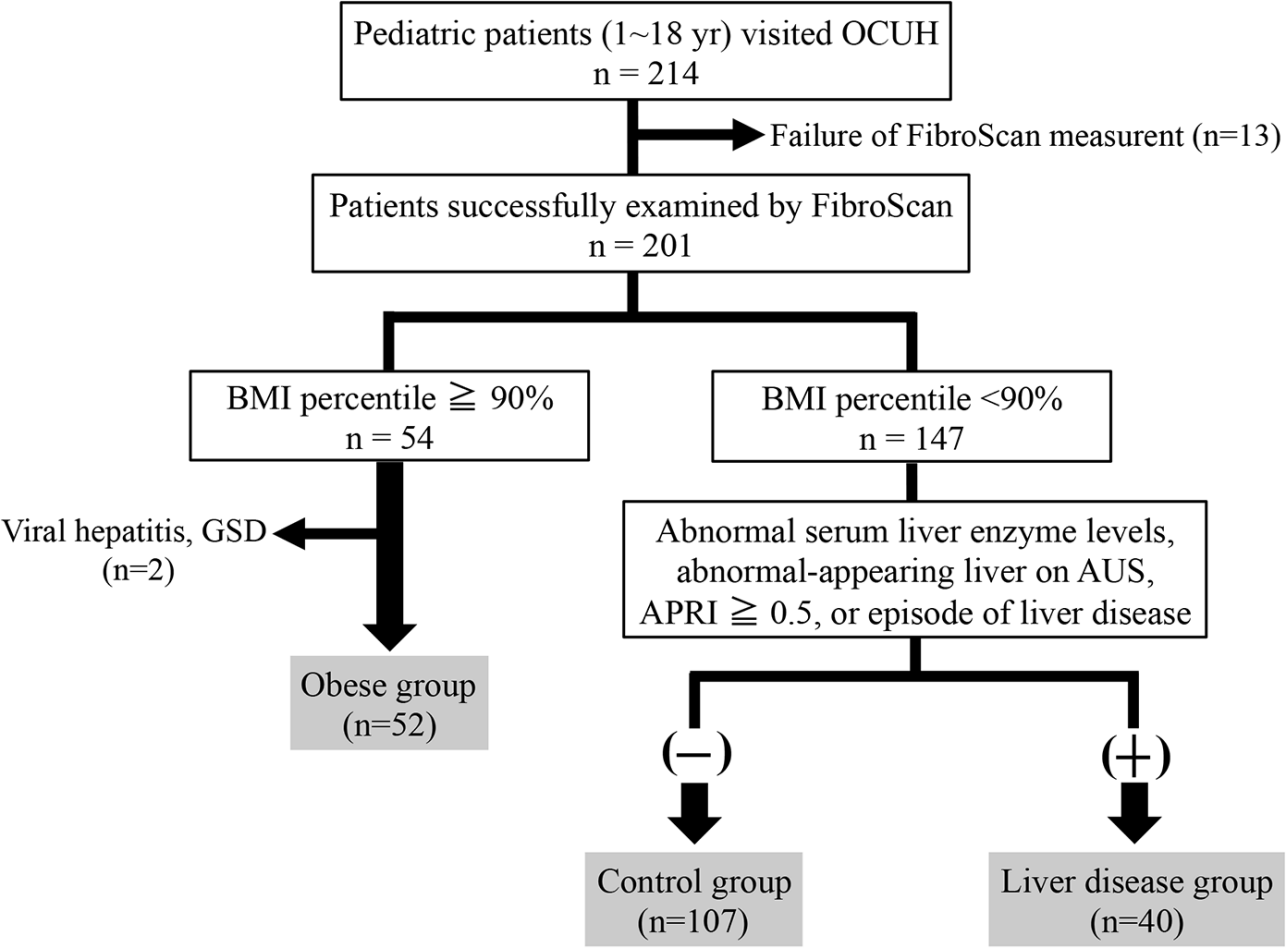
Diagram of the study population selection process. Patients who were examined by using FibroScan at a success rate of greater than 60% with 10 valid measurement and an interquartile range (IQR) of 30% or less than 30% of the median LSM value were analyzed for the study. Patients whose body mass index (BMI) was at the 90^th^ percentile or higher and without underlying liver diseases were included in the obese group. Patients whose BMI was lower than the 90^th^ percentile were divided into 2 groups: control group or liver disease group. The control group was defined as having normal serum liver enzyme levels, an aspartate aminotransferase (AST)-to-platelet ratio index (APRI) score below 0.5, a normal-appearing liver on abdominal ultrasonography, and no episodes of liver disease. The remaining patients were included in the liver disease group.

The study population comprised pediatric patients (age, 1–18 years) who were examined by using FibroScan at the Department of Pediatrics at Osaka City University Hospital (Osaka, Japan) between April 2013 and August 2014. Patients in whom FibroScan evaluations were successful according to the criteria described later were divided into three groups: obese group; liver disease group; and control group (Fig 1). The obese group comprised patients whose body-mass index (BMI) was in the 90^th^ percentile or higher and who lacked an underlying liver disease. Among patients whose BMI was lower than the 90^th^ percentile, control subjects were defined as having normal serum liver enzyme levels, an aspartate aminotransferase (AST)-to-platelet ratio index (APRI) score below 0.5, a normal-appearing liver on abdominal ultrasonography (AUS), and no episodes of liver disease (Fig 1). Because APRI is a reliable negative predictive value for advanced liver disease [14,15,29,30], we included it in the determination of the control group. The remaining non-obese pediatric patients were defined as the liver disease group (Fig 1).

The Osaka City University Graduate School of Medicine Ethics Committee specifically approved this study (the approved number: 2610). Written informed consent was obtained from each child’s parent or legal guardian, and written assent was obtained from children who were at least 6 years old, after children and each child’s parent or legal guardian were given, in writing, a full explanation of the aims of the study, its possible hazards, discomfort, and inconvenience. The ethics committee approved this consent procedure.

### Evaluation of liver stiffness measurement (LSM) and controlled attenuation parameter (CAP)

Values for LSM, a marker for liver fibrosis, and CAP, an indicator of hepatic fat deposition, were obtained by using FibroScan according to the manufacturer’s instructions [4]. All patients were evaluated by using the 3.5-MHz standard M probe (diameter, 7 mm), which can be used to measure both LSM and CAP. Although FibroScan’s S probe (5 MHz; diameter, 5 mm) is designed for small children [14], it can only be used to measure LSM. In addition, our preliminary study comparing the difference in LSM value between S and M probe demonstrated no significant difference (S1 Table). Because our study focused on the significance of simultaneous determination of LSM and CAP in children, we used the M probe in all of the pediatric patients in this study.

With the patient lying in the dorsal decubitus position, AUS was used to identify a region of the liver that was free of large vascular structures; the tip of the FibroScan transducer was then placed on the skin between the ribs over the right lobe of the liver, and the LSM value was recorded. The final LSM result was expressed in kilopascals (kPa) and was the median value of 10 individual valid measurements. CAP is a measure of the attenuation of ultrasound waves in the liver at 3.5 MHz that was measured at the same time as LSM by using the M probe. The final CAP value, which ranges from 100 to 400 decibels per meter (dB/m), is the median of 10 individual valid measurements. For each patient, the success rate was calculated as the ratio of the number of successful measurements to the total number attempted (expressed as a percentage). An examination was considered successful when 10 valid measurements with a success rate of at least 60% were taken and the interquartile range (IQR) was 30% or less than 30% of the median LSM value. Subjects with unsuccessful examinations were excluded from the analyses.

### Clinical and biochemical parameters

Clinical parameters (height, body weight, and BMI) were collected on the same day that the FibroScan examination was performed. Biochemical parameters (serum AST, aspartate aminotransferase [ALT], triglycerides [TG], total cholesterol [T-Cho], hyaluronic acid [HA], and type IV collagen 7S [7S collagen] concentrations and APRI score) were measured within 3 months before or after the FibroScan examination [1,5]. APRI score was calculated by using the following formula: (AST / upper limit of normal × 100) / platelet count [29,30].

### Liver ultrasonography

Liver ultrasonography was performed and evaluated by three experienced ultrasonographers. Fatty liver was diagnosed when the patient had a fatty liver infiltration score of 3 or greater on a scale of 6, as described previously [20]. Briefly, the infiltration score was the sum of the subscores for the following 3 criteria: (1) The contrast between the liver and kidney parenchyma (L–K contrast). Patients received a subscore of 2 when the L–K contrast was markedly increased, a subscore of 1 when there was a slight increase, and a subscore of 0 when there was a homogenous echo level and unclear L–K contrast; (2) The attenuation of echogenicity in the deep regions of the liver (deep attenuation). Patients received a subscore of 2 when deep attenuation was marked, a subscore of 1 when deep attenuation was modest, and a subscore of 0 when deep attenuation was absent; and (3) The blurring of liver blood vessel structures (vascular blurring). Patients received a subscore of 2 when vascular blurring was pronounced, a subscore of 1 when vascular blurring was moderate, and a subscore of 0 in the absence of vascular blurring.

### Liver histology

A subset of 8 patients underwent liver biopsy. Formalin-fixed and paraffin-embedded sections of biopsy specimens were stained with hematoxylin and eosin stains and Azan stain. The histologic features of the liver biopsies were scored according to the NAFLD activity scoring system proposed by Kleiner et al. [31] as follows: 0, no fibrosis; 1, periportal or perisinusoidal fibrosis; 2, perisinusoidal and portal or periportal fibrosis; 3, bridging fibrosis; and 4, cirrhosis. Fibrosis yielding a score of 2 or greater was considered clinically significant. Liver biopsy samples were then classified as definitive NASH, borderline NASH, or simple steatosis, as described [15].

### Statistical analyses

The 3 groups were compared by using the Kruskal–Wallis test for continuous variables. When the Kruskal–Wallis test identified a significant difference (*P* < 0.05), the Mann–Whitney U test with Bonferroni correction was used to identify the source of difference. Two-sided *P*-values < 0.05 were considered significant. Correlations between variables were described by using Spearman’s rank correlation coefficient (*ρ*). Spearman’s *ρ* values > 0.4 were considered indicative of positive correlation. Multivariate linear regression analyses were performed to identify factors affecting liver stiffness.

## Results

### FibroScan is feasible irrespective of age in children

**Fig 2:**
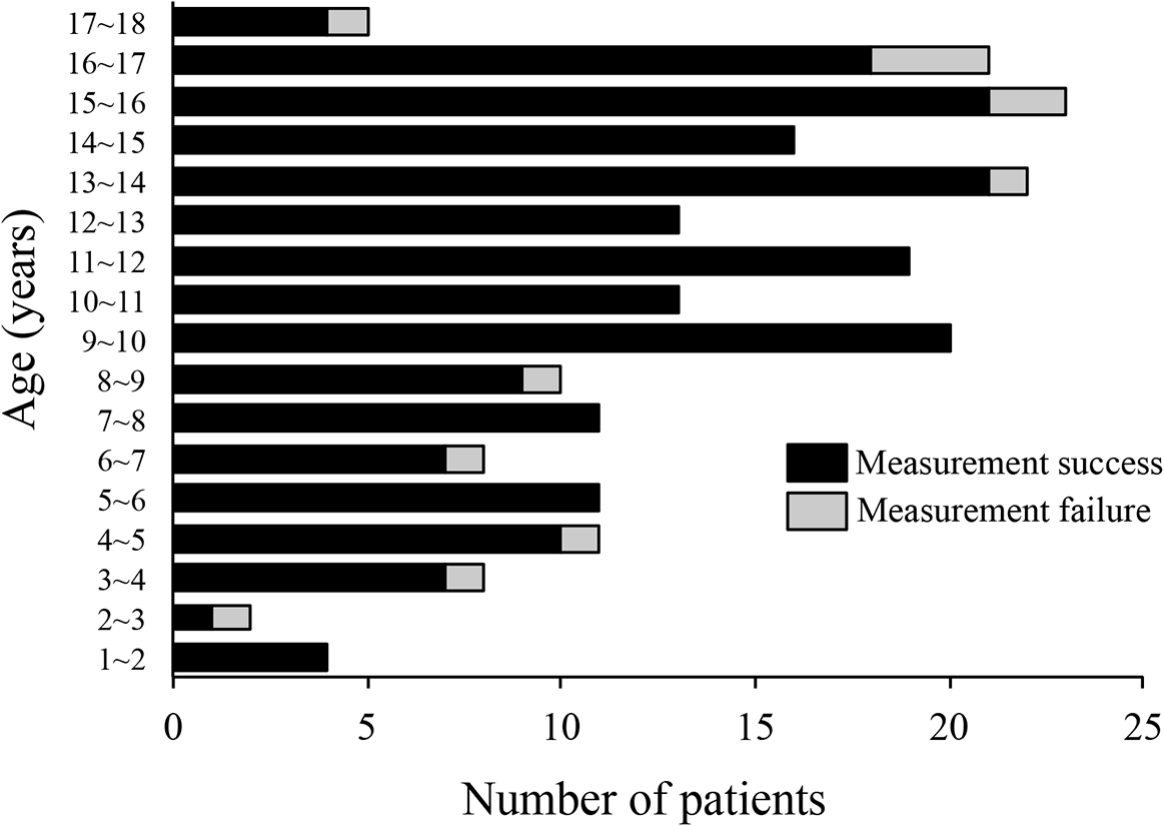
Age distribution of the patients examined by using FibroScan. A total of 214 children and adolescents (age, 1.3–17.6 years; 121 male) were examined by using FibroScan. A total of 201 patients (93.9%; median age, 11.5 years; range, 1.3–17.6 years) (black bars) were examined successfully; the evaluation was unsuccessful in the remaining 13 patients (6.1%; median age, 13.1 years; range, 2.0–17.2 years) (gray bars) due to excessive thickness of subcutaneous adipose tissue in 5 obese children (BMI percentile [mean ± 1 SD], 99.6 ± 2.2), poor cooperation in 4 young children (2.0, 3.7, 4.0, and 6.2 years), and no obvious reason in 4 non-obese and cooperative children.

Among the 214 children (1.3–17.6 years; 121 male) examined, 201 children (93.9%; median age, 11.5 years; range, 1.3–17.6 years; 115 male) were successfully evaluated by using FibroScan (Figs 1 and 2). Evaluations were unsuccessful in the remaining 13 children (6.1%; median age, 13.1 years; range, 2.0–17.2 years; 6 male) (S2 Table) due to excessive thickness of subcutaneous adipose tissue in 5 obese children (BMI percentile [mean ± 1 SD], 99.6 ± 2.2), poor cooperation in 4 young children (2.0, 3.7, 4.0, and 6.2 years), and no obvious reason in 4 non-obese and cooperative children (Figs 1 and 2 and S2 Table). These results demonstrate that FibroScan evaluation is feasible in children of all ages.

### Childhood and adolescent obesity is associated with elevated levels of AST, ALT, TG, and APRI

The clinical and biochemical profiles of each group are summarized in Table 1. AST, ALT, TG, and APRI values were significantly (*P* < 0.001 for AST, ALT and TG; *P* = 0.006 for APRI) higher in obese patients compared with the control group; these parameters did not differ between the obese and liver disease groups (Table 1). HA and 7S collagen concentrations were not statistically different among the three groups (Table 1).

**Table 1:** Characteristics of the 199 patients in the study.

	All	Control group	Obese group	Liver disease group	Kruskal-Wallis test, *P* value	Mann- Whitney U test, *P* value		
						Control vs Obese	Control vs Liver disease	Obese vs Liver disease
n	199	107	52	40				
Median age, yr (range)	11.5 (1.3–17.6)	11.2 (1.3–17.0)	13.0 (3.5–17.6)	10.7 (1.6–16.7)	0.02	0.04	*NS*	*NS*
Male / female	115 / 84	54 / 53	38 / 14	23 / 17	*NS*			
Median BMI percentile (range)	62.08 (0–99.98)	50.69 (0–89.44)	97.14 (90.00–99.98)	39.32 (0–88.51)	< 0.001	< 0.001	*NS*	< 0.001
AST*, IU/L	38 ± 34	24 ± 7	48 ± 45	53 ± 43	< 0.001	< 0.001	< 0.001	*NS*
ALT*, IU/L	42 ± 59	17 ± 14	75 ± 83	49 ± 58	< 0.001	< 0.001	< 0.001	*NS*
TG*, mg/dL	130± 146	97 ± 65	149 ± 73	163 ± 296	< 0.001	< 0.001	*NS*	*NS*
APRI*	0.467± 0.46	0.28 ± 0.08	0.59 ± 0.61	0.70 ± 0.52	< 0.001	0.006	< 0.001	*NS*
HA*, ng/mL	36 ± 154	20 ± 16	15 ± 9	108 ± 336	*NS*			
7S collagen*, ng/mL	4.1 ± 2.6	3.8 ± 0.7	3.8 ± 0.7	5.5 ± 5.5	*NS*			
Viral hepatitis, n	3	0	0	3				
Non-viral hepatitis, n	3	0	0	3				
Liver tumor, n	1	0	0	1				
GSD, n	10	0	0	10				
Wilson disease, n	1	0	0	1				
PSC, n	1	0	0	1				
Portal hypertension, n	1	0	0	1				
Diabetes mellitus, n	18	5	12	1				
Kidney disease, n	19	16	3	0				
Metabolic disease, n	25	18	4	3				
Neurological disease, n	17	11	4	2				
Thyroid disease, n	6	5	1	0				
Endocrine disease, n	12	10	1	1				
Others, n	49	31	5	13				
No underlying disease, n	33	11	22	0				

BMI, body mass index; AST, aspartate transaminase; ALT, alanine aminotransferase; TG, triglycerides; APRI, aspartate transaminase to platelets ratio index; HA, hyaluronic acid; 7S collagen, collagen type IV; GSD, glycogen storage disease; PSC, primary sclerosing cholangitis; NS, not significant

The 3 groups were compared by using the Kruskal–Wallis test for continuous variables. When the Kruskal–Wallis test identified a significant difference (*P* < 0.05), the Mann–Whitney U test with Bonferroni correction was used to identify the source of difference. Two-sided *P*-values < 0.05 were considered significant.

*Results are expressed as mean ± 1 SD.

### FibroScan results correlate highly with histologic and ultrasonographic grading

**Fig 3:**
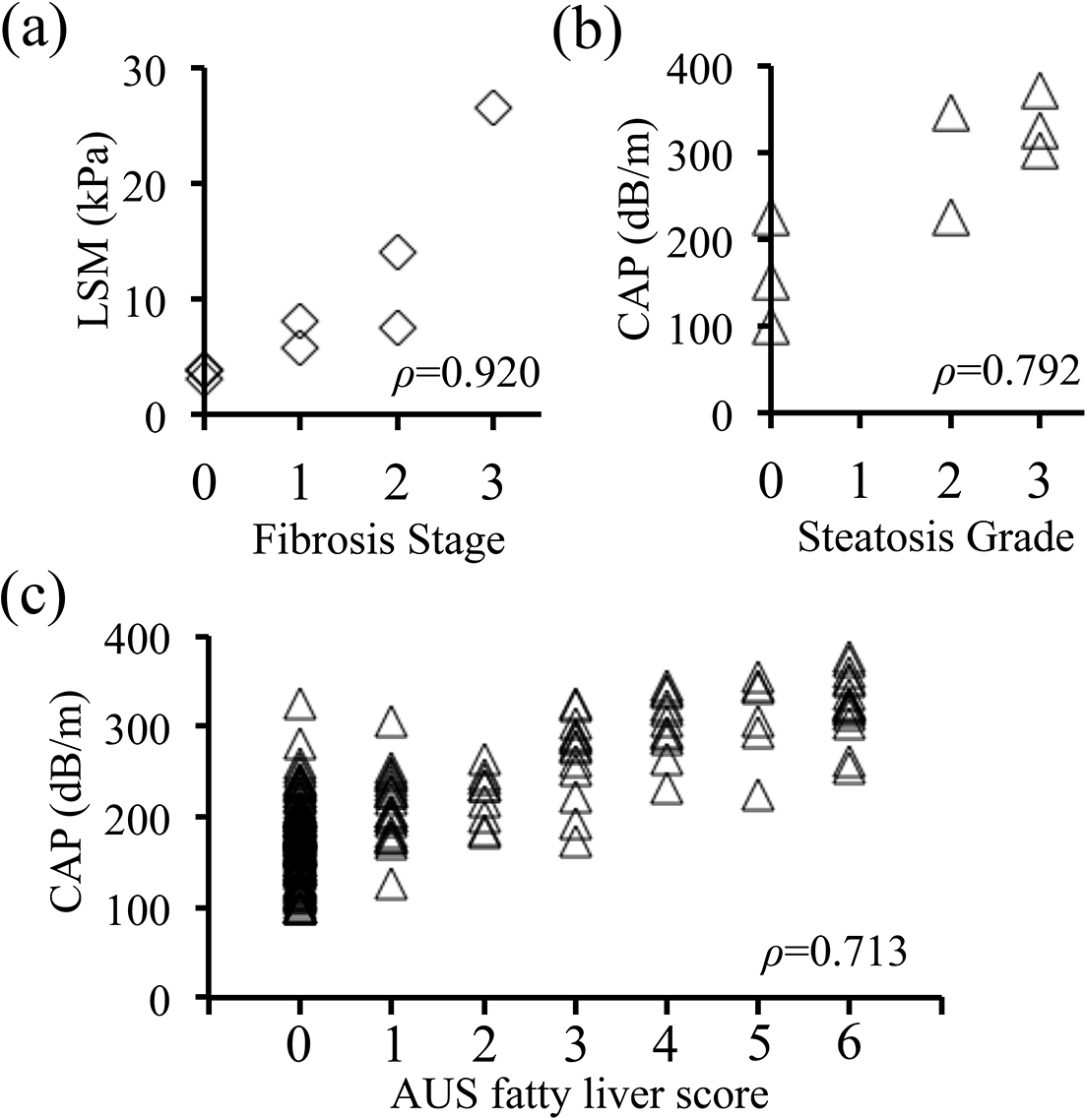
Comparison of results from FibroScan, liver biopsy, and abdominal ultrasonography (AUS). a and b. Liver stiffness measurement (LSM) and controlled attenuation parameter (CAP) values from FibroScan evaluations were compared with histologic fibrosis stage and steatosis grade. Liver biopsy was performed in 8 pediatric patients, in which the underlying disease was simple obesity in 4 patients, type C hepatitis in 2 patients, type B hepatitis associated with obesity in 1 patient, and liver transplantation for treatment of congenital biliary atresia in 1 patient. Among the 5 obese patients, four patients were diagnosed with NASH, and the remaining patient was diagnosed with simple steatosis. a. Correlation between LSM value and histologic fibrosis stage. LSM was highly correlated with fibrosis stage (Spearman’s *ρ* = 0.920). b. Correlation between CAP value and histologic steatosis grade. CAP value was highly correlated with steatosis grade (*ρ* = 0.792). c. Correlation between CAP and fatty liver infiltration score calculated according to AUS findings. CAP was highly correlated with AUS fatty liver infiltration score (*ρ* = 0.713).

Although the reliability of FibroScan has been well studied in adults [1–11], its reliability specifically in children needed to be verified. We therefore compared the FibroScan results with the histologic findings and AUS fatty liver infiltration scores of our pediatric study population (Fig 3). Because of the invasiveness of the procedure, only 8 of our subjects (S3 Table) underwent liver biopsy. The underlying conditions in this patient subset were simple obesity in 4 patients, type C hepatitis in 2 patients, type B hepatitis associated with obesity in 1 patient, and liver transplantation for treatment of congenital biliary atresia in 1 patient (S3 Table). Among the 5 obese patients (CAP, 314 ± 56 dB/m; LSM, 7.9 ± 3.9 kPa), 4 patients (CAP, 337 ± 30 dB/m; LSM, 8.9 ± 3.6 kPa) were diagnosed with NASH; the remaining patient (CAP, 225 dB/m; LSM, 3.8 kPa) was diagnosed with simple steatosis (S3 Table). Comparison of the biochemical data, the FibroScan data and histologic assessment revealed that HA and 7S collagen were mildly correlated (*ρ* = 0.690 and *ρ* = 0.423, respectively) with the fibrosis stage, whereas the LSM value was highly correlated (*ρ* = 0.920) with the fibrosis stage and the CAP value was highly correlated (*ρ* = 0.792) with the steatosis grade (Fig 3). In addition, the CAP values were highly correlated (*ρ* = 0.713) with the fatty liver infiltration score calculated from AUS findings (Fig 3).

### Obese children have more liver stiffness and fat deposition than do non-obese children

**Fig 4:**
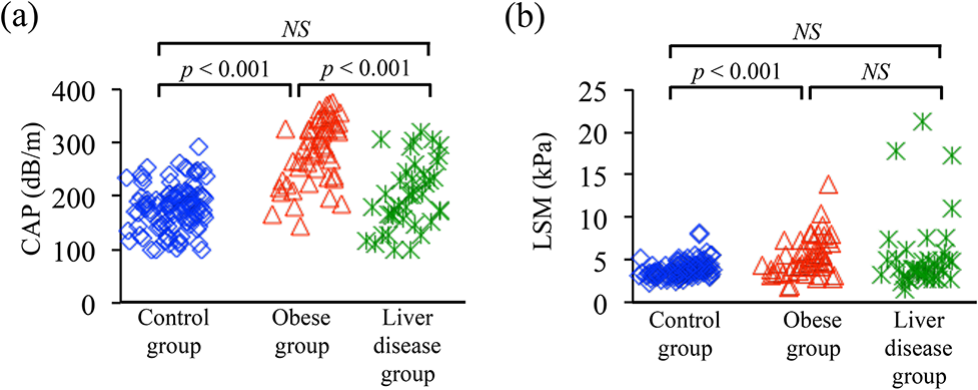
Comparison of CAP values among control, obese, and liver disease groups. a. CAP was significantly higher in the obese group (mean ± 1 SD, 285 ± 60 dB/m) compared with both the control group (179 ± 41 dB/m; *P* < 0.001) and the liver disease group (202 ± 62 dB/m; *P* < 0.001). b. LSM was significantly higher in the obese group (5.5 ± 2.3 kPa) than in the control group (3.9 ± 0.9 kPa, *P* < 0.001) but did not differ between the liver disease group (5.4 ± 4.2 kPa) and either the obese or control group.

**Fig 5:**
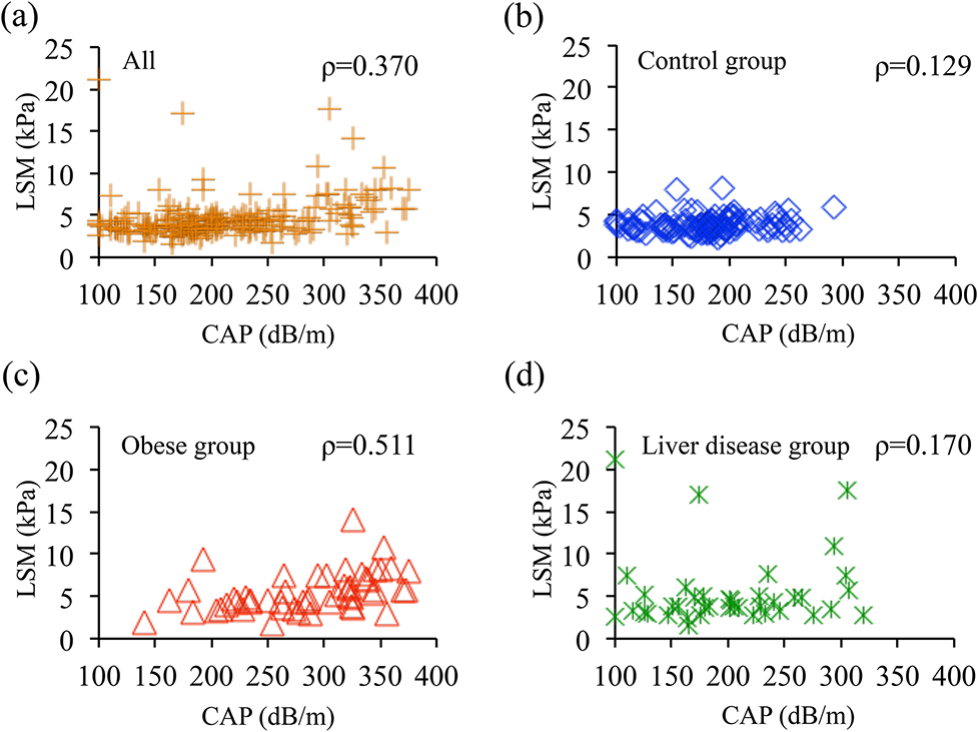
Correlation between LSM and CAP values. LSM was positively correlated with CAP in the obese group (Spearman’s ρ = 0.511) but not in the control (ρ = 0.129) or liver disease (ρ = 0.170) group.

The CAP value was significantly higher in the obese group (285 ± 60 dB/m) than in both the control group (179 ± 41, *P* < 0.001) and the liver disease group (202 ± 62, *P* < 0.001) (Fig 4). The LSM value was significantly higher in the obese group (5.5 ± 2.3 kPa) than in the control group (3.9 ± 0.9, *P* < 0.001), but there were no significant differences in LSM between the liver disease group (5.4 ± 4.2) and either the obese or control group (Fig 4). When the correlation of CAP and LSM was evaluated among the 3 groups, LSM was highly correlated with CAP in the obese group (*ρ* = 0.511) but did not show significant correlation in the control (*ρ* = 0.129) or liver disease (*ρ* = 0.170) group (Fig 5). These results indicate that childhood obesity is highly associated with hepatic fat deposition and liver stiffness and that hepatic fat deposition correlates with liver stiffness in the obese pediatric population.

### ALT correlates positively with LSM and CAP in obese children

Among the biochemical markers shown in Table 2 (AST, ALT, TG, T-Cho, APRI, 7S collagen, and HA), the CAP value was positively correlated (*ρ* = 0.446) with ALT in the obese group but was not significantly correlated with any biochemical marker in either the control or liver disease group. The LSM value was positively correlated with AST (*ρ* = 0.730), ALT (*ρ* = 0.772), and APRI (*ρ* = 0.788) in the obese group but was not significantly correlated with any biochemical marker in either the control or liver disease group. In multivariate analysis, APRI, age, BMI percentile, and ALT levels were the independent predictors of liver stiffness in all subjects, whereas ALT and age were the independent predictors of liver stiffness in the obese group (S4 Table).

**Table 2:** Correlation of FibroScan results and biochemical values.

	CAP				LSM			
	All	control group	obese group	liver disease group	All	control group	obese group	liver disease group
AST	0.217	-0.045	0.382	0.058	0.202	-0.262	0.730	-0.106
ALT	0.440	0.127	0.446	0.250	0.329	-0.015	0.772	-0.076
TG	0.434	-0.020	0.190	0.366	0.183	-0.128	0.043	0.153
T-cho	0.095	-0.286	0.291	0.395	-0.060	-0.305	0.181	-0.057
APRI	0.209	0.107	0.330	-0.013	0.293	-0.045	0.788	-0.005
7S collagen	-0.151	0.037	-0.177	-0.453	0.131	0.121	0.198	0.211
HA	-0.009	0.174	0.055	0.014	0.082	0.079	0.145	0.214

Correlation between CAP or LSM and AST, ALT, TG, T-cho, APRI, 7S collage or HA, were evaluated in each group using Spearman rank correlation coefficients (ρ). CAP, controlled attenuation parameter; LSM, Liver stiffness measurement; AST, aspartate aminotransferase; ALT, alanine aminotransferase; TG, triglycerides; T-Cho, total cholesterol; APRI, aspartate aminotransferase-to-platelet ratio index; 7S collagen, type IV collagen 7S; HA, hyaluronic acid

## Discussion

FibroScan simultaneously obtains both LSM and CAP data, thus achieving rapid quantification of hepatic fibrosis and steatosis, respectively [4,5] and allowing noninvasive discrimination between simple steatosis and NASH in patients with NAFLD. However, the suitability of FibroScan for use in the pediatric population and reliable indicators for its application remained to be clarified because fewer studies have been conducted in children than in adults [12–15]. The present study clarified the feasibility of FibroScan in Japanese children. In addition, we disclosed that Japanese obese children, particularly those with elevated ALT levels, are at risk for both hepatic steatosis and fibrosis and therefore are good candidates for FibroScan screening.

The first important finding of the current study is that Japanese obese children and adolescents showed increased CAP and LSM values compared with those in non-obese subjects as well as significant correlation of these values. Because liver stiffness correlates with the histologic stage of liver fiborosis, our results indicate that childhood obesity is highly associated with hepatic fat deposition and fibrosis and that hepatic fat deposition is correlated with liver fibrosis in the obese pediatric population. Therefore, FibroScan offers the advantage of simultaneously determining CAP and LSM values and thus may play an important role in hepatic evaluations, especially in obese children. In contrast, LSM values in the obese population need to be interpreted carefully, because several factors in addition to liver fibrosis influence liver stiffness [32, 33]. In other studies, liver stiffness increased with the central venous pressure in patients with congestive heart failure [32], and the LSM was mildly elevated in patients with steatohepatitis, perhaps secondary to inflammation [33]. Obesity in the children in our study was highly associated with hepatic fat deposition and increased serum aminotransferase. Because steatohepatitis might affect the LSM values in an obese population, the significance of the LSM value in obese children merits investigation in a future study.

The second important aspect of our current findings is the value of the ALT measurement prior to FibroScan screening. Our results showed that, although childhood and adolescent obesity were associated with elevated levels of AST, ALT, TG, and APRI, only ALT positively correlated with both LSM and CAP values in the obese group. Because NAFLD is the major chronic liver disease of obese children, effective screening for NAFLD is an important task in the management of obesity. In this regard, an elevated ALT level is the primary laboratory abnormality in patients with NAFLD [34], but not all patients with NAFLD have elevated levels of ALT [26,35], and the diagnostic sensitivity of serum ALT for NASH is only about 40% [36]. Together our current results and previous findings indicate that ALT is an important biomarker that is suggestive of but not diagnostic of hepatic steatosis and fibrosis. Therefore, although FibroScan screening to evaluate liver stiffness and steatosis is a priority for obese children with elevated ALT levels, this imaging method should be considered even for obese children with normal ALT concentrations.

Another important feature of FibroScan is its feasibility in children. Our study showed that FibroScan can be performed highly successfully in patients representing a wide age range (1 to 18 years). However, 6.1% of measurements were invalid in 214 consecutive examinations. Previous studies using FibroScan have reported a failure rate of 5%~11% in adults [11, 37, 38], and one pediatric study [14] reported a failure rate of 15% in healthy children. Factors independently associated with FibroScan measurement failure in adult subjects include female sex, high BMI, and metabolic syndrome [11]. In addition, BMI and central obesity were independent risk factors for unreliable LSM in Chinese adult patients [38]. In a pediatric study [14], the highest failure rate was recorded for children younger than 6 years.

In our study, 2 important reasons for measurement failure were the excessive thickness of the subcutaneous adipose tissue in obese adolescents and the poor cooperation of young children (2.0, 3.7, 4.0, and 6.2 years). Because patients must be immobile during FibroScan imaging, we speculate that young children may require sedation for the procedure. In addition, because the region of interest for the measurement of liver stiffness with the M probe lies 2.5 cm below the skin surface, excessively thick subcutaneous adipose tissue is an important risk factor for measurement failure. In this regard, the differences in body composition associated with different ethnicities may influence obesity-induced measurement failure, and additional studies should be accumulated. Overall, previous findings and our current results indicate that FibroScan methodology is feasible in pediatric subjects of diverse ages, but poor cooperation in young children and the degree of obesity should be weighed as risk factors for measurement failure.

The ‘gold standard’ for the evaluation of NAFLD and chronic hepatitides B and C is a liver biopsy [26]; however, this painful, invasive procedure is associated with several complications and therefore is unsuitable for routine screening or repeated examination in children. Alternatively AUS is a first-line screening tool for fatty liver [20,26,39,40], but results can vary depending on the skill of the examiner. Although computed tomography (CT) and magnetic resonance imaging (MRI) are both reliable techniques for the evaluation of hepatic steatosis [41,42], CT exposes the patient to radiation, and both modalities are time-consuming and expensive, and require sedation. Furthermore, there is no consensus regarding the quantitative assessment of liver fibrosis, although a few studies have shown potential benefits of CT or MRI [43,44]. Given that non-invasiveness is a key feature of diagnostic modalities for children, the non-radiative and painless characteristics of FibroScan combined with its applicability to a broad age range make this methodology particularly suitable for pediatric patients and may encourage its use by pediatricians.

Our study has several limitations. First, because we were unable to collect a large number of cases in which liver biopsy was performed, we were unable to define cut-off values of LSM and CAP for determining the degree of liver fibrosis and hepatic fat deposition. However we were able to verify high correlation between FibroScan parameters and liver histology scores (Fig 3). A second limitation is that the subjects in our study were not enrolled from the general population but from pediatric patients who visited our hospital; therefore FibroScan results may differ between the control patients in our study and healthy individuals in general population. In addition, liver disease group comprised of patients with very different etiologies or no underlying hepatologic disease, thus the etiology-based characteristics of FibroScan results is uncertain in liver disease group. However our study focused on differences in the liver profile between obese patients and non-obese patients with or without liver disease, and revealed that FibroScan screening should focus on the liver stiffness for the non-obese patients with liver disease in spite of the etiology, whereas screening should focus on the evaluation of both liver stiffness and steatosis for the obese patients. Our findings have contributed to showing the usefulness of FibroScan screening in a hospital-based pediatric population.

In conclusion, the present study revealed that FibroScan was feasible irrespective of age in Japanese children. Therefore FibroScan imaging, which provides simultaneous determination of CAP and LSM, is a non-invasive and effective screening method for hepatic steatosis and liver fibrosis in Japanese obese children, particularly those with elevated serum ALT.

## Supporting Information

S1 Table Comparison of LSM values obtained by using the S and M probes. (DOCX)

S2 Table Profiles of the 13 patients with measurement failure. (DOCX)

S3 Table Profiles of the 8 patients who had liver biopsy. (DOCX)

S4 Table Multivariate regression analysis for factors associated with liver stiffness. (DOCX)
