# Supplementary material for: Transient Elastography-Based Liver Profiles in a Hospital-Based Pediatric Population in Japan
Source: PLoS One. 2015 Sep 23;10(9):e0137239. doi: 10.1371/journal.pone.0137239 (PMC4580651; doi:10.1371/journal.pone.0137239)
Supplement: S1 Table — (DOCX) [file pone.0137239.s001.docx]

Supplemental table 1. Comparison of LSM value between S and M probe.

|  | M probe (Mean±SD, kPa) | S probe (Mean±SD, kPa) | *P* value* |
| --- | --- | --- | --- |
| 1~5 yr (n=18) | 4.6±3.32 | 5.4±5.75 | 0.741 |
| 6~10 yr (n=25) | 5.4±6.90 | 4.5±1.22 | 0.497 |

For pediatric use, FibroScan has two probes (S and M) that differ in impulse power, diameter, and measurement depth. The S probe (5 MHz; diameter, 5 mm) is designed for small children, and can only be used to measure LSM. The M probe (3.5 MHz; diameter, 7 mm) is designed for school children and adolescent, and can be used to measure both LSM and CAP. In 43 children, LSM was measured twice – once with the S probe and one with the M probe. Patients were divided into two groups according to age: preschool (1 – 5 years old; n=18) and school children (6 – 10 years old; n=25). There was no statistical difference in LSM value between M and S probe. * Comparison between M and S probe was performed with the Mann-Whitney U test. Two-sided *P*-values < 0.05 were considered significant.
