# Supplementary material for: Transient Elastography-Based Liver Profiles in a Hospital-Based Pediatric Population in Japan
Source: PLoS One. 2015 Sep 23;10(9):e0137239. doi: 10.1371/journal.pone.0137239 (PMC4580651; doi:10.1371/journal.pone.0137239)
Supplement: S2 Table — (DOCX) [file pone.0137239.s002.docx]

Supplemental table 2. Profile of 13 patients with measurement failure.

| No. | Gender | Age  (years) | Height  (cm) | Weight  (kg) | BMI | BMI percentile | Reason for measurement failure |
| --- | --- | --- | --- | --- | --- | --- | --- |
| 1 | M | 2.0 | 85.3 | 12.3 | 16.9 | 81.9 | poor cooperation |
| 2 | F | 3.7 | 100 | 15.4 | 15.6 | 60.2 | poor cooperation |
| 3 | F | 4.0 | 90.5 | 14.2 | 17.3 | 90.9 | poor cooperation |
| 4 | F | 6.2 | 110.5 | 18.9 | 15.5 | 51.3 | poor cooperation |
| 5 | M | 8.5 | 133 | 28.2 | 16.0 | 48.7 | unknown |
| 6 | F | 9.5 | 120 | 24.7 | 17.2 | 61.8 | unknown |
| 7 | F | 13.1 | 148 | 69.3 | 31.6 | 99.6 | obesity |
| 8 | M | 15.4 | 169 | 76.9 | 26.9 | 94.9 | obesity |
| 9 | F | 15.7 | 164 | 65 | 24.2 | 85.4 | unknown |
| 10 | M | 16.3 | 175 | 162 | 53.1 | 100.0 | obesity |
| 11 | M | 16.4 | 149 | 74.6 | 33.8 | 99.3 | obesity |
| 12 | F | 16.8 | 155 | 37.5 | 15.7 | 51.3 | unkwon |
| 13 | M | 17.2 | 185 | 154.1 | 45.0 | 99.9 | obesity |

A success rate was calculated as the ratio of the number of successful measurements to the total number attempted (expressed as %). An examination was considered successful when 10 valid measurements with success rate of at least 60% were taken and interquartile range (IQR) was 30% or less than 30% of the median LSM value. Subjects with measurement failure were excluded from the analyses.

BMI, body mass index
