# Supplementary material for: Transient Elastography-Based Liver Profiles in a Hospital-Based Pediatric Population in Japan
Source: PLoS One. 2015 Sep 23;10(9):e0137239. doi: 10.1371/journal.pone.0137239 (PMC4580651; doi:10.1371/journal.pone.0137239)
Supplement: S3 Table — (DOCX) [file pone.0137239.s003.docx]

Supplemental table 3. Profile of 8 patients who had liver biopsy.

| Age  (yr) | Gender | Underlying disease | Fibrosis  stage^1^ | Steatosis  grade^2^ | LSM  (kPa) | CAP  (dB/m) | AST  (IU/L) | ALT  (IU/L) | HA  (ng/ml) | 7S collagen  (ng/ml) |
| --- | --- | --- | --- | --- | --- | --- | --- | --- | --- | --- |
| 13.0 | M | obesity | 1C | 2 | 8.1 | 345 | 83 | 154 | 10.0 | 4.3 |
| 15.3 | M | obesity | 1C | 3 | 5.8 | 373 | 31 | 56 | 10.7 | 3.0 |
| 16.6 | M | obesity | 2 | 3 | 14.1 | 326 | 241 | 356 | 33 | 4.3 |
| 11.1 | M | obesity | 2 | 3 | 7.5 | 302 | 97 | 173 | 48 | 4.1 |
| 9.4 | M | HBV / obesity | 0 | 2 | 3.8 | 225 | 24 | 26 | <10 | 3.6 |
| 14.7 | F | HCV | 0 | 0 | 3.8 | 151 | 22 | 17 | 17 | 3.9 |
| 6.2 | M | HCV | 0 | 0 | 3.0 | 225 | 43 | 35 | 26 | 4.4 |
| 10.5 | M | Post-liver transplant^3^ | 3 | 0 | 26.6 | 100 | 66 | 47 | 97.6 | 6.6 |

*^1^ Fibrosis stage: 0; None, 1;Perisinusoidal or periportal, 1A; Mild, zone 3, perisinusoidal, 1B Moderate, zone 3, perisinusoidal, 1C; Portal/peroportal, 2; Perisinusoidal and portal/ periportal, 3; Bridging fibrosis, 4; Cirrhosis

*^2^ Steatosis grade:Low- to medium-power evaluaton of parenchymal involvement by steatosis 0; <5%, 1; 5-33%, 2; >33-66%, 3; >66% *^3^ The patient had liver transplantation for the congenital biliary atresia.
