# Supplementary material for: Transient Elastography-Based Liver Profiles in a Hospital-Based Pediatric Population in Japan
Source: PLoS One. 2015 Sep 23;10(9):e0137239. doi: 10.1371/journal.pone.0137239 (PMC4580651; doi:10.1371/journal.pone.0137239)
Supplement: S4 Table — (DOCX) [file pone.0137239.s004.docx]

Supplemental table 4. Multivariate regression analysis for factors associated with liver stiffness

| Characteristic | | Coefficient (β) | *t* | 95% CI for β | *P* value |
| --- | --- | --- | --- | --- | --- |
| All subjects | |  |  |  |  |
|  | APRI | 3.592 | 5.164 | 2.218 – 4.965 | <0.0001 |
|  | Age | 0.185 | 4.079 | 0.096 − 0.275 | 0.0001 |
|  | BMI percentile | 0.012 | 2.112 | 0.001 – 0.023 | 0.036 |
|  | ALT | -0.011 | -1.980 | -0.022 − 0.000 | 0.049 |
| Obese group | |  |  |  |  |
|  | ALT | 0.016 | 5.179 | 0.001 – 0.002 | <0.0001 |
|  | Age | 0.196 | 2.905 | 0.060 – 0.331 | 0.006 |
|  | BMI percentile | 0.142 | 1.445 | -0.056 – 0.340 | 0.154 |

ALT, alanine aminotransferase; APRI, aspartate aminotransferase-to-platelet ratio index; BMI, body mass index
